# Neuroinflammation in Huntington’s disease: From animal models to clinical therapeutics

**DOI:** 10.3389/fimmu.2022.1088124

**Published:** 2022-12-22

**Authors:** Qingqing Jia, Shihua Li, Xiao-Jiang Li, Peng Yin

**Affiliations:** Guangdong Key Laboratory of Non-human Primate Research, Guangdong-Hongkong-Macau Institute of CNS Regeneration, Jinan University, Guangzhou, China

**Keywords:** Huntington’s disease, immune response, inflammation, animal models, immune therapies

## Abstract

Huntington’s disease (HD) is a progressive neurodegenerative disease characterized by preferential loss of neurons in the striatum in patients, which leads to motor and cognitive impairments and death that often occurs 10-15 years after the onset of symptoms. The expansion of a glutamine repeat (>36 glutamines) in the N-terminal region of huntingtin (HTT) has been defined as the cause of HD, but the mechanism underlying neuronal death remains unclear. Multiple mechanisms, including inflammation, may jointly contribute to HD pathogenesis. Altered inflammation response is evident even before the onset of classical symptoms of HD. In this review, we summarize the current evidence on immune and inflammatory changes, from HD animal models to clinical phenomenon of patients with HD. The understanding of the impact of inflammation on HD would help develop novel strategies to treat HD.

## 1 Introduction

Huntington’s disease (HD) is a devastated neurodegenerative disorder caused by a trinucleotide CAG repeat expansion in exon 1 of HD gene encoding for huntingtin (HTT) protein (1–3). The normal *HTT* gene contains less than 36 CAG repeats, whereas mutant *HTT* carriers 40 or more CAG repeats with complete penetrance. Expansions varying from 36 to 39 CAG repeats may also result in HD, but with incomplete penetrance (1). The CAG repeat expansion encodes an expanded polyglutamine (polyQ) tract in mutant HTT that elicits neuronal loss in HD (4). One of the important mechanisms for neurodegeneration in HD is the inflammation caused by mutant HTT, which is the focus of this review for discussion. Generally, mutant HTT causes the inflammatory response in both of the central nervous system (CNS) and peripheral tissues directly. Immunomodulatory messengers can cross the blood-brain barrier (BBB) in both directions, with inflammation spreading from the periphery to the CNS or vice versa (5, 6). The degree of microgliosis and astrogliosis correlates with disease severity directly, and result in an increased production of inflammatory mediators. The increase in microglial activation seemed to be a very early event that occurs before the neuropathological changes. Pathological processes underlying HD, such as excitotoxicity or oxidative stress, induce the morphological change of microglia by a constant expression of inflammatory mediators (7). The secreted pro-inflammatory cytokines can damage nerve cells, leading to their death (8). Astrocytes are also susceptible to inflammatory processes in the brain, becoming activated locally by dead neurons or pathogens. For example, the mutant HTT expressed in glial cells, primarily astrocytes in HD mouse models, could reduce their neuroprotective function (9, 10). However, in some conditions, the neuroinflammation can benefit neuronal tissue by promoting cell debris clearance (11). This review will summarize the growing evidence on inflammatory changes, including the pros and cons of pathogenic mutant HTT, from HD animal models to patients’ clinical phenotypes. The better understanding of the impact of neuroinflammation cytokines on HD will open new venues for the development of novel therapeutic targets.

## 2 Neuroinflammation in different animal models of Huntington’s disease

### 2.1 Drosophila models

Drosophila melanogaster has been used as an early HD animal model to identify metabolic biomarkers at pre-symptomatic and symptomatic stages of the disease. *In vivo* experiments have revealed that the ectopic overexpression of mutant human HTT (exon 1 with expanded CAG repeats) in the neural tissue of transgenic flies causes neurodegeneration (12, 13). However, there was limited work on neuroinflammation in Drosophila model of HD. Lin et al. observed that the expression of mutant HTT (Q93) in hemocytes did not directly affect the animal survival, but the numbers of circulating hemocytes were significantly decreased, leading to an impaired immune response against pathogenic invasion. Mutant HTT caused the altered production of cytokines, such as *upd3*, *dome*, *tep1, totA, totB, and totC*, and consequently the immune dysregulation in Drosophila (14).

### 2.2 Mouse models

Identification of the genetic mutation for HD has accelerated the establishment of various transgenic or knock-in HD mouse models, in which human mutant HD gene is inserted randomly into the mouse genome or precisely into the endogenous mouse *HTT* gene. In HD transgenic mice, the animals expressed a full-length or a fragment of the mutant *HTT* gene in addition to the two normal copies of the endogenous mouse *HTT* gene (15). Common transgenic models of HD are divided as follow. Firstly, N-terminal HTT fragment models with expanded CAG-repeats, such as R6/2, R6/1 or N171-82Q, present an earlier onset of motor and cognitive abnormalities (16). The R6/1 and R6/2 transgenic mice were the first transgenic model developed to study HD, which express exon 1 of the human HD gene with around 115 and 150 CAG repeats respectively (16). Secondly, full-length transgenic mouse models carry mutant HTT in a yeast artificial chromosome (YAC) or bacterial artificial chromosome (BAC), which present a slow progression of the disease with comparable neuropathology and motor-related behavior changes (17, 18). Despite the wealth of different transgenic HD mice available currently, the R6/2 and YAC128 mouse strains are widely used to study many pathological aspects of HD (19). Symptoms of motor impairment in R6/2 mice appear around 3-6 weeks of age. A continuous loss of weight results in death between 11-14 weeks of age (20–22). In YAC128 mice with its full length mutant HTT spanning about 120 CAG repeats (18), hypoactivity is first seen until the age of 8 months. Additionally, progressive gait abnormalities, ataxia, hind limb clasping, and a progressive decline in the forced motor function occur over time (23). In HD knock-in mice, the pathological CAG-repeat is integrated into the mouse *HTT* gene in a homozygous or heterozygous manner. Because knock-in mice carry the mutation in its appropriate genetic and protein context, they are the most faithful genetic model of human disease. The widely used HdhQ150 and zQ175 mouse models exemplarily belong to this group (24–26). Knock-in mice exhibit more slow progression and age-dependent development of behavioral, pathological, cellular, and molecular abnormalities, which makes these animals valuable for studying age-dependent neuropathology and symptoms.

Based on the various transgenic or knock-in HD mouse models, many researchers reported the important role of the immune system in HD. Träger et al. investigated the myeloid cells from different HD mouse models of R6/2, HdhQ150 and YAC128, to assess whether they are similarly hyperresponsive as HD patient cells. They found that blood CD11b+ cells isolated from 12-week-old R6/2 and 22-month-old HdhQ150 are hyper-reactive upon LPS stimulation *in vitro*, replicating the phenotype observed in human cells. Analyzing the cytokine profile in 3-month-old YAC128 mice, peritoneal macrophages were found to produce high levels of IL-6 upon the standard endotoxin stimulation. These mouse models recapitulate altered cytokine profiles identified in HD patients, and therefore they could be used to model the immune phenotype (27). Pido-Lopez et al. revealed that the increased TNF-α and IL-12 levels in the striatum of 14-week-old R6/2 mice while IL-12 and IL-1β decreased in the cortex (28). In contrast, Godavarthi et al. found no differences in cytokine expression, including MCP-1, IL-6, IL-10, TNF-α, interferon-γ and IL-12, in the brain samples of R6/2 mice compared with the age-matched control. However, the Iba-1 immunostaining did not reveal any significant increase in the numbers of microglia in the cortex and striatum of R6/2 mice at 12 weeks, an age when these mice show severe symptoms (29). Recently, the fractalkine signaling axis has been explored in the 8-20 weeks old R6/1 HD mouse, and the expression levels of cx3cl1 have been significantly decreased (30), which may lead to an increase in synaptic pruning mediated by microglia, as the total density of PSD-95 puncta decreased and the density of PSD-95 puncta in Iba1 stained microglia increased significantly (30). Furthermore, administering CX3CL1 protein was able to rescue R6/1 mice from long-term depression. It appears that striatal synaptic plasticity dysfunction in pre-symptomatic R6/1 mice is caused by a reduction in CX3CL1 (30), which is consistent with the previous research that CX3CL1 regulates microglia engulfment of synapse (31). Additionally, quinolinic acid (QUIN) has been associated with neuroinflammation in various neurological diseases (32). It is possible that the activation of microglia and astroglia may result in a production of the excitotoxic metabolites, such as the kynurenine pathway, before morphological markers of glial activation are apparent (33–35). Thus, abnormalities in the kynurenic acid pathway are an interesting aspect of HD pathology. QUIN and 3-hydroxykynurenine (3-HK) are neurotoxic metabolites that are elevated in the cortex of HD patients at the early stages of disease progression (36). Especially the 3-HK levels were elevated in the striatum, cortex and cerebellum in the R6/2 starting at 4 weeks of age significantly and selectively, and both 3-HK and QUIN levels were increased in the striatum and cortex in Hdh^Q92^ and Hdh^Q111^ at 15 months and YAC128 mouse at 8 months (35).

The altered immune system function, not only in the CNS but also peripherally, has been implicated in HD pathogenesis (6, 37). To elucidate peripheral inflammatory activation in HD, Björkqvist et al. measured the levels of multiple cytokines in the serum of R6/2, Hdh150Q/150Q and YAC128 mice. In 12-week-old R6/2 mice, IL-6, IL-10, IL-1β, and IL-12p70 were significantly increased as compared to control animals. However, the IL-6, IL-10, and IL-12p70 were significantly elevated in 22-month-old Hdh150Q/150Q mice. In the YAC128 mouse model of HD at 12 months of age, the similar elevations in serum IL-6 and IL-8 were observed (6). Similary, Chang et al. detected the levels of inflammatory markers in the plasma of R6/2 mice. IL-6 levels in R6/2 mice at different disease stages (9, 11 and 13 weeks) were higher than those in the age-matched wild-type (WT) littermates, with higher levels of MMP-9 and TGF-β1 compared with their WT littermates from 11 weeks. In contrast, the plasma level of IL-18 (11-13 weeks old) was lower than control mice (38). Furthermore, Disatnik et al. also measured the levels of inflammatory cytokines such as TNF and IL-6, the levels of both these cytokines were two times higher in the plasma of 13 weeks old R6/2 mice than WT mice (39). Interestingly, treatment with P110 (a selective peptide inhibitor, of excessive mitochondrial fission) for 8 weeks was efficient in reducing the levels of these inflammatory levels to the level in WT mice (39, 40). A study including 9-12 months old YAC128 mice serum samples revealed the significant increases in IL-8 and IL-10 levels. However, the elevation of IL-6 in serum of YAC128 mice occurred at 12 months of age whereas the level of IFN-γ increased in serum samples from YAC128 mice were at 6-9 months old (41). More recently, Podlacha et al. also reported the significant elevation of levels of inflammatory markers, such as IL-6, TNF-α, IL-1β and IL-12, in R6/1 mouse peripheral blood. This allows for a more objective assessment of particular biomarkers in course of a slower progression of symptoms in HD mice. At early stages of disease, the anti-inflammatory defense mechanism was significantly impaired, with a marked decrease in IL-10 levels (42). Valadão et al. also investigated the immune changes in many peripheral organs of the 12-month-old BAC HD model. They found significant changes in cytokine levels in all organs analyzed, including heart, liver, spleen and kidney. Levels of IL-6 and IL-12p70 were increased in the heart of BAC HD mice as compared with WT animals. In the liver samples of BAC HD mice, enhanced IL-12p70 and TNF-α levels were observed. In the spleen, there was an increase in the levels of the cytokine IL-4, but a decrease in the levels of IL-5 and IL-6 in BAC HD mice. However the increased level of IL-6 was exhibited in kidney (43).

Considering that pro-inflammatory cytokine IL-6 may actively influence the disease course in HD, an excessive IL-6 release was also detected in YAC128 mice at 12 months and R6/2 mice at 9, 11 and 13 weeks of age (6, 38). Bouchard et al., administered an IL-6 neutralizing antibody to R6/2 mice to test whether IL-6 influences the disease course. In their work, treatment with the specific antibody in R6/2 mice reduced weight loss at late stages and partially rescued motor deficits on the rotarod performance as compared with the control IgG treatment (44). Following this line of reasoning, Wertz et al. tested the hypothesis that IL-6 deficiency would be protective against the effects of mutant huntingtin and therefore generated the R6/2 mice model lacking IL-6. Contrary to previous studies, the lack of IL-6 exacerbated R6/2 associated behavioral phenotypes. Based on a single nuclear RNA sequencing of striatal cell types, it was evident that IL-6 deficiency affected normal regulation of various genes associated with synaptic function, as well as the BDNF receptor Ntrk2 (45).

Although relatively shorter experimental period and lower housing cost of using rodents are the advantages of studying mouse models of HD, the data on activation of the inflammatory system in HD mouse models are sparse and contradictory, possibly due to the considerable pathological differences between different transgenic strains and at different disease stages. The immunocytochemical staining of GFAP or Iba-1 has been widely used to identify gliosis, an early CNS damage that is associated with neuroinflammation in HD (46–49). Different levels of gliosis activation were observed in mouse models with full-length or a fragment HTT at different ages. Yu et al. systematically compared that the N171-82Q mouse striatum and found a GFAP increase, at 3 months of age and became prominent at 4-5 months of age, indicated by intense labeling throughout astroglial cell bodies and their fibrous processes. Although the striatum of R6/2 mice at 12 weeks of age displayed some increased GFAP staining, the overall number of GFAP-labeled glial cells was not increased in R6/2 mice compared with that of their littermates or other HD mice. In R6/1 mice even at 9 months of age, there was still little GFAP staining in the striatum (50). Reiner et al. also compared the cortex (22 and 30 weeks) and striatum (30 weeks) of R6/2 chimeras, which displayed some glial cells with increasingly upregulated GFAP staining, much later than pure R6/2 mice that died at 12-15 weeks of age (51). Gatto et al. found that the neuroinflammatory processes with activated astrocyte GFAP occurred in the 11- and 30-week-old R6/1 mice (52). In 17-week-old R6/2 mouse model, reactive astrocytes with processes enveloping degenerating neurons was seen, though no accompanying inflammatory response or increased macrophages and microglia was observed (53). Simmons et al. also reported that the Iba-1 positive cells did not obviously differ in both R6/2 and WT mice at the age of <7 weeks, but increased in R6/2 mice older than 8 weeks (54). While Hdh150Q knock-in mice at 27-30 weeks of age also did not show intense GFAP immunoreactivity, a significant increase in GFAP immunoreactivity was found in the striatum of Hdh^150Q^ mice at 14 months of age, as reported previously (26, 50). In HD KI mice expressing shorter CAG repeats (HdhQ92 or HTTQ111), no gliosis was found up to 17-18 months of age (55, 56). However, in 18-20 months old Hdh175/175 mouse, the expression of full-length mHTT in microglia was able to promote pro-inflammatory transcription (57). Different extents of gliosis seen in HD mice are likely associated with genetic background, CAG repeats in the HD gene, housing and experimental conditions. However, the data in HD patients show that the inflammatory response is activated at the very early disease stage (6). It is also necessary to point out that most of rodent models fail to mimic the overt and typic neurodegeneration seen in HD patients (58–60). The lack of overt neuronal loss and robust gliosis in HD mouse models prevents the rigorous evaluation of the therapeutic effects on neurodegeneration. The summarized evidence on gliosis activation, including the pros and cons observations, was listed in **Table 1**.

**Table 1:** Changes in glial cells in different HD mouse models at multiple ages.

Model	Age	Glial status	Reference
R6/2	2-16 weeks	Iba-1 positive cells did not obviously differ in both R6/2 and wild-type mice at 2-4 and 5-7 weeks, but increased at 8-10 and 11-13 weeks old in R6/2 mice.	(54)
R6/2	4 and 13 weeks	Microglia increased significantly in the 4 and 13 weeks old R6/2 mice compared to the age-matched WT.	(61)
R6/2	8-12 weeks	R6/2 striatal revealed increased Iba-1^+^ microglia and GFAP^+^ astroglia at a symptomatic stage of 12 weeks old.	(62)
R6/2	12 weeks	Reactive astrocytes were rarely observed in the cortex and striatum of HD and microglia did not change in number at 12 weeks old R6/2.	(29)
R6/2	22 and 30 weeks	GFAP was increasingly upregulated in the cortex (22 and 30 weeks), and striatum (30 weeks) of R6/2 chimeras much later than pure R6/2 mice that died at 12-15 weeks of age.	(51)
R6/1	11 and 30 weeks	GFAP, an astrocyte activated markers, was found in the early stage of the disease in R6/1 mice.	(52)
R6/1	9 months	Little GFAP staining in the striatum in R6/1 mice at 9 months of age.	(50)
N171-82Q	3-5 months	GFAP staining increased in the striatum of N171-82Q mice at 3 months of age and became prominent at 4-5 months of age.	(50)
YAC128	3, 6 and 12 months	Microglia increase in both size and number in YAC128 mice at 12 months old.	(63)
KI 140Q	4,12 and 23 months	GFAP immunostaining showed no change in KI mice at 4 months but increased at >12 months.	(64)
Hdh150Q	27-30 weeks and 42-52 weeks	Hdh150Q mice at 27-30 weeks of age did not show intense GFAP immunoreactivity but a marked increase in GFAP immunoreactivity in the striatum of 42-52-week-old Hdh150Q.	(26)
Hdh150Q	14 months	14-month-old HdhCAG150 mice had a greater number of GFAP-positive cells.	(50)
HttQ111	2-18 months	HttQ111/+ mice at 18 months of age did not show increased gliosis.	(56)
HdhQ175	18-20 months	Expression of full-length mHTT in microglia was able to promote pro-inflammatory transcription at 18-20 months of age.	(57)

### 2.3 Large animal models

The biological differences between humans and mice may account for the failure of some mouse models to replicate pathology seen in humans. Thus, it is possible that larger transgenic animal models may be able to mimic important neurodegenerative features. However, an HD transgenic sheep model (OVT73) was created, which does not exhibit many of the overt phenotypes observed in HD patients and was thought to be a model of prodromal or early-stage HD (65). By using CRISPR/Cas9, Yan et al. established the 140Q KI pig that endogenously expresses full-length mutant HTT (66). Importantly, the increased immunohistochemical staining of GFAP was firstly observed in the dorsal caudate nucleus and putamen of the 4-5-month-old F0 KI and F1 KI pigs. Also, immunostaining with the antibody to Iba-1 revealed a marked increase of the microglial cells in KI pig brain, which is more abundant in the striatum than in the cortex. Quantification of the number of different types of cells revealed that the KI striatum had the most severe loss of NeuN-positive cells and the highest increase in glial cell numbers, which was not observed in the age-matched HD KI mouse (66, 67). Additionally, Valekova et al. used transgenic HD minipig, which was generated by injecting lentiviral vectors carrying truncated mutant huntingtin genes that encode 124 glutamine repeats integrated into chromosome 1q24-q25 and transmitted through successive three generations (68), for investigating multiple cytokines (69). At the same time, various cytokines were analyzed in the secretomes of microglia and blood monocytes, as well as in the cerebrospinal fluid (CSF) and serum collected from pre-symptomatic HD minipigs. A decline in IFN-α was observed in CSF collected from an early time at 9 months of age and lasted at least up to 36 months. The transgenic minipigs at 36 months of age had lower levels of IL-10 in the CSF. IFN-α and IL-10 levels were also decreased in secretome of microglia, whilst elevated IL-8 and IL-1β levels were secreted by primary microglia that were isolated from the HD minipigs at 36 months of age. In serum samples collected from 36-month-old HD minipigs had significantly higher levels of IL-8 than WT ones (69).

**Figure 1:**
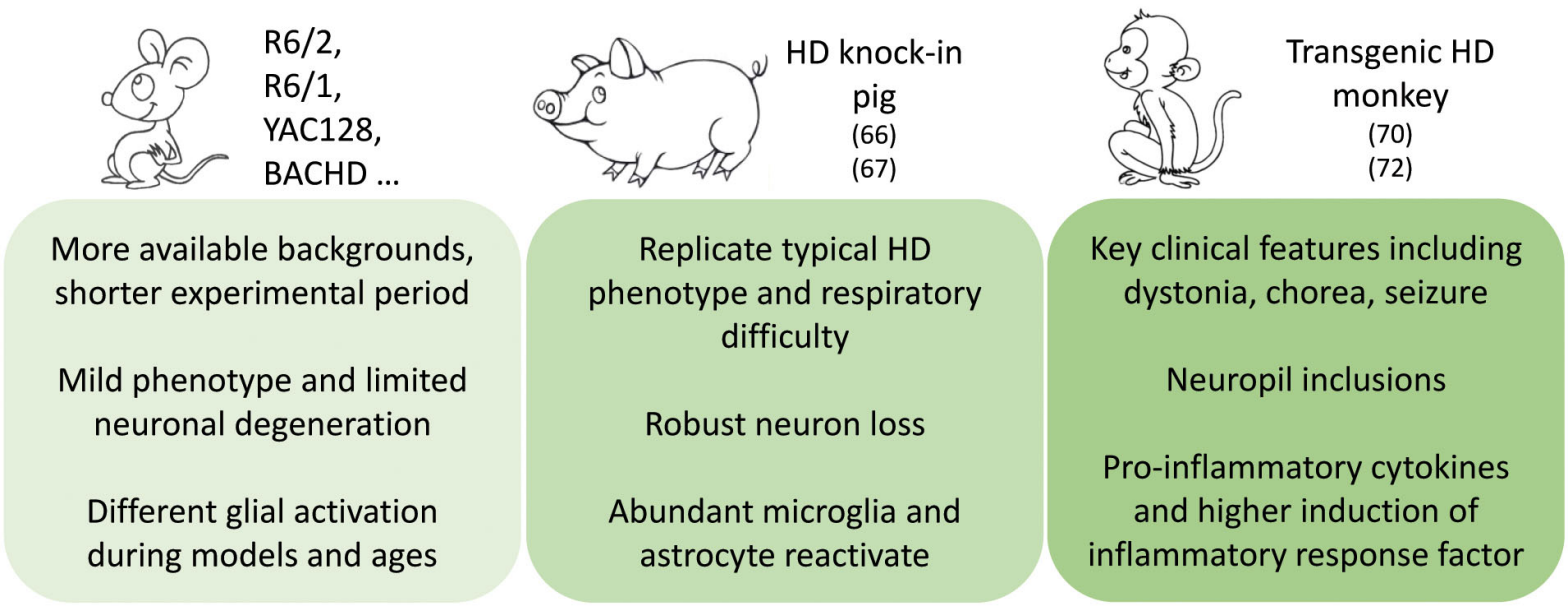
Pathogenic insights from and phenotype differences between animal models expressing mutant HTT.

Besides, transgenic non-human primate models expressing the disease genes were established, transgenic HD rhesus monkeys, which express exon 1 mutant HTT with 84Q under the control of the human ubiquitin promoter, were generated by injecting lentiviruses into fertilized oocytes to express mutant HTT (70). HD transgenic monkeys with 84Q die postnatally, and this early death was associated with the overexpression of N-terminal mutant HTT. Despite their early death, some transgenic monkeys developed key clinical HD features including dystonia, chorea, and seizure (70). Interestingly, the 5-year-old transgenic HD monkeys expressing N-terminal HTT 509 amino acids with approximately 67-72Q under the human *HTT* promoter (71) exhibited increased pro-inflammatory cytokines and higher induction of immune pathway genes, such as inflammatory response factor IL-6, TNF-α, and C-Reactive Protein (CRP) (72) (**Figure 1**).

## 3 Neuroinflammation in Huntington’s disease patients

Robust evidence regarding neuroinflammation in HD comes from postmortem studies of brain tissues from patients with HD. Evidence for involvement of inflammation in the pathogenesis of HD includes upregulation of inflammatory cytokines or chemokines and activation of the complement system. Björkqvist et al. have reported the increase of multiple cytokines in the plasma (IL-1β, IL-4, IL-6, IL-8, TNF-α, IL-10) and striatum (IL-6, IL-8 and TNF-α) of HD patients (6). Interestingly, a significant increase in IL-6 plasma levels was found in pre-symptomatic HD mutation carriers 16 years prior to the predicted onset of the disease, suggesting that inflammatory changes occurs very early in the disease process (6). According to Dalrymple et al., the level of IL-6 in HD patient plasma was increased significantly (37). Likewise, HD patients also exhibit increased expression of IL-6, IL-8, and matrix metalloproteinase-9 (MMP-9) in their cortex and cerebellum (73). Remarkably, MCP1/CCL2 and IL-10 mRNA levels were significantly higher in the striatum of HD patients than controls (73). Gang et al. also quantified the plasma concentrations of IFN-γ, IL-1β, IL-2, IL-4, IL-6, IL-8, IL-10, IL-12p70, IL-13, and TNF-α from HD patients. Patients with HD have significantly lower plasma concentrations of IL-4, a marker of responses from T-helper-2 cells, than healthy controls. In contrast, no significant differences was observed in the plasma concentrations of IFN-γ, IL-1β, IL-2, IL-6, IL-8, IL-10, IL-12p70, IL-13 or TNF-α in HD patients (74). Recently, von Essen MR et al. reported the immune abnormalities before motor onset of disease (75). Proinflammatory cytokines, including IL-17, were detected in the CSF of HTT mutation carriers, as well as the increased IL-7 consumption before motor onset of HD. Moreover, they reported an increased prevalence of IL-17 producing Th17.1 cells in the CSF of HTT mutation carriers, predominantly in pre-motor manifest individuals. There was a negative correlation between intrathecal Th17.1 cell frequency and the progression of HD, suggesting that Th17.1 cells play an important role at the early stages of the disease. Moreover, the author found that the balance of pro-inflammatory and regulatory T cells was skewed. This skewing further favors a pro-inflammatory environment in the CSF of HTT mutation carriers (75).

In particular, a significant elevation of chemokines (C-C motif) ligand (CCL)-2, CCL4, CCL11, CCL13, CCL26 has been detected in the plasma from HD patients (76). Wild et al. have also reported that CCL11 and related chemokines may directly contribute to the neurodegenerative processes in CNS. As a result of analyzing plasma levels of cytokines in two separate cohorts of HD patients, the elevated levels of CCL11 and CCL26 were found in HD group from their first cohort of 65 HD patients (76). There were also significant differences in CCL11 and CCL26 levels across all HD clinical stages. In their second cohort of 68 HD patients and 26 healthy controls, plasma CCL11 and CCL26 levels were significantly increased with more advanced HD cases. In addition, CCL11 levels were positively correlated with standardized assessments of motor impairment while negatively correlated with functional scores (76, 77). Thus, it is worth performing future studies of both CCL11 and CCL26 as potential biomarkers in HD. It has been shown that the complement factors C1QC, C2, and C3 are also increased in CSF samples taken from living HD patients in comparison with controls (78). Singhrao et al. also found the complement activation, and increased mRNA levels of complement proteins in HD brains (79). Increased protein expression of complement components C7 and C9, complement inhibitor cluster proteins, and acute phase protein α-2-macroglobulin were shown in HD patient plasma and CSF (37).

**Figure 2:**
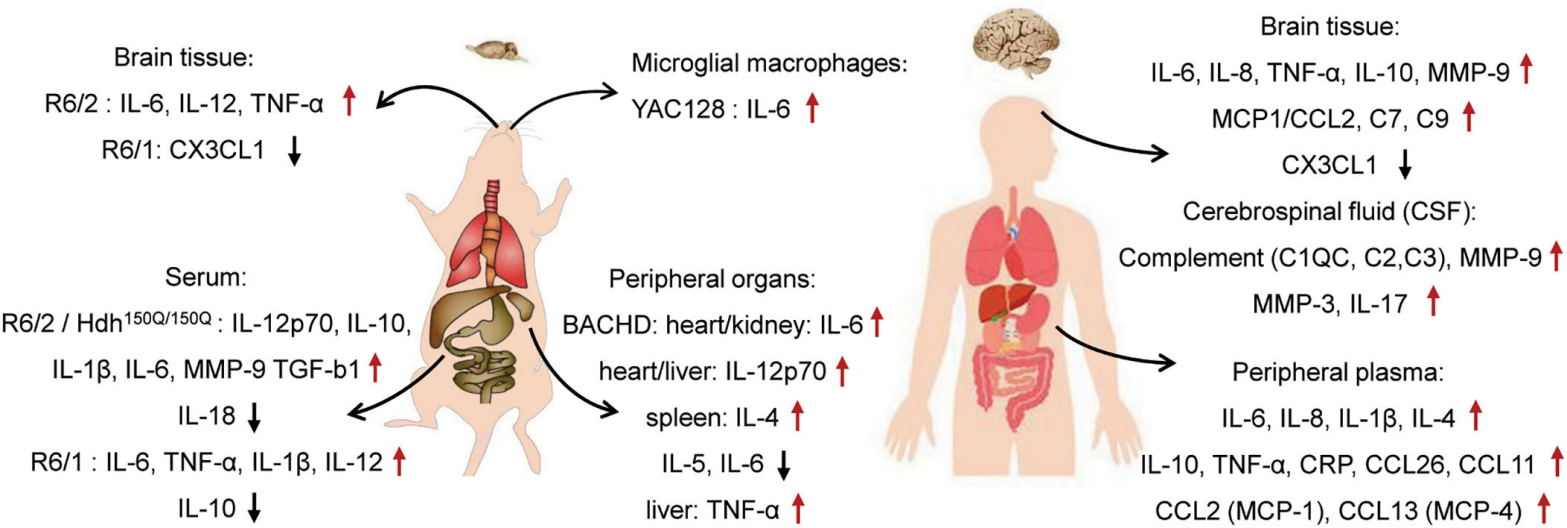
Schematic summary of brain and peripheral inflammatory changes in HD mice and patients.

Neuroinflammation can be modulated by neuron-glial signaling through various soluble factors, such as cluster of differentiation CD22 (80), CD47 (81), CD200 (82, 83), the family of CD300 receptors (84) and CX3CL1 (85). CX3CL1 produced in neurons is the sole member of the CX3C family of chemokines (86), and it is known that CX3CL1 is the only ligand binding to CX3CR1, a 7 transmembrane domain class A G-protein coupled receptor that is expressed in microglia, monocytes, natural killer cells (NK), T cells and smooth muscle cells (87). Interestingly, CX3CL1 was also found to be an important novel factor in HD pathogenesis and survival following a network analysis of microarray data from human post-mortem tissue (88). An investigation of the fractalkine signaling axis in HD patients revealed that cx3cl1 gene expression was significantly reduced in their putamen (30) (**Figure 2**).

## 4 Immune therapies

The accumulating evidences supports that inflammation plays a key role in neurodegenerative diseases and has stimulated the use of immunotherapeutic strategies to modulate neuroinflammatory diseases. Several preclinical and clinical trials of potential immunomodulatory drugs have been investigated in HD, such as minocycline and cannabinoids, laquinimod, TNF-α inhibitors, anti-SEMA4D monoclonal antibody, gangliosides and so on.

### 4.1 Minocycline and cannabinoids

It has also been demonstrated that minocycline and cannabinoids have anti-inflammatory properties, even though they are not a member of the classical definition of anti-inflammatory drugs. R6/2 mice were administered with the anti-inflammatory tetracycline minocycline showed improvements in behavioral and neuropathological deficits (89, 90), which is also supported by an excitotoxic rat model of HD (91). A clinical study revealed that minocycline at 100 and 200 mg/day was well tolerated for 8 weeks. The study involved 60 patients who were randomly assigned to receive placebo (n = 23), minocycline 100 mg/day (n = 18), or minocycline 200 mg/day (n = 19). However, there was no effect on the UHDRS (Unified HD Rating Scale) scores, with no efficacy observed (92). Cannabidiol was considered safe and well tolerated during a 6 weeks clinical trial conducted on 15 patients affected by HD. However, clinical outcomes did not show the significant improvement (93).

### 4.2 Laquinimod

The immunomodulator laquinimod (LAQ) could downregulate both the production of pro-inflammatory cytokines in peripheral blood mononuclear cells and neuroglial activation in the brain, which was initially known as an immunomodulatory agent and was used to treat multiple sclerosis. For example, the neuroprotective effect of LAQ is well supported by its therapeutic effects on animal models of neuroinflammatory diseases, such as the experimental autoimmune encephalomyelitis (EAE) (94, 95), and by alleviation of demyelination in different diseases (96–99). LAQ has also been shown to improve behavioral phenotypes and white matter integrity in many HD mouse models (100–104). Especially, LAQ rescues evidence of cortico-striatal neurodegeneration, demyelination of white matter, and behavioral deficits in YAC128 HD mice (105). A mild ameliorative effect of LAQ was also observed in R6/2 mice with motor function deficits and striatal neuropathology (106). Thus, the therapeutic effect of LAQ is therefore thought to be due to an anti-inflammation effect (98, 103). Although the precise mechanism of action of LAQ is unclear, Dobson et al. demonstrated that LAQ significantly dampened the release of hyper-reactive cytokines from stimulated premanifest and manifest HD patient monocytes, and may exert its neuroprotective effects by promoting BDNF production (94). However, LAQ had no efficacy observed in the Phase 2 clinical trials of HD patients unfortunately. (https://clinicaltrials.gov/ct2/show/NCT02215616). Thus, whether LAQ can be used to treat HD patients remains to be verified.

### 4.3 TNF-α inhibitor

TNF-α is a multifunctional cytokine associated with cellular proliferation, differentiation, inflammation, immune responses and apoptosis (107). Based on the evidence of increased levels of TNF-α in HD, one study investigated the therapeutic potential of DN-TNF-α (XPro1595), which demonstrated that intracerebroventricular (ICV) injection of DN-TNF-α modulates neuroinflammation, caspase activation, mHTT aggregate burden, and motor function deficit in R6/2 HD transgenic mice (108). However, the clinical efficacy of this molecule in human HD warrants further investigation based on the fact that DN-TNF-α’s systemic injection rather than an ICV injection shows lesser efficacy on motor function in R6/2 mice (109). However, in a study of R6/2 mice carried out by Pido-Lopez et al., the systemic injection of etanercept, a drug that inhibits TNF-α, dampened the levels of TNF-α in plasma and other peripheral proinflammatory molecules such as IL-1β and IL-6. However, the level of TNF-α and IL-6 expression in the striatum is not affected by etanercept treatment. According to the follow-up study, etanercept partially reduced brain atrophy, but failed to improve HD related functional and cognitive deficits in R6/2 mice (110).

### 4.4 SEMA4D antibody

Semaphorin 4D (SEMA4D), also called CD100, is chemorepulsive axonal guidance and immunoregulatory transmembrane signaling molecule. It signals *via* three receptor subtypes of Plexin-B1 (PLXNB1), Plexin-B2 (PLXNB2) and CD72. In the CNS, SEMA4D interacts with PLXNB1 in neuronal cells *via* Rho-GTPases-RhoA and R-Ras GTPase-activating protein activities, inducing axonal growth cone collapse (111, 112). In previous studies, the treatment on YAC128 HD transgenic mice with neutralizing SEMA4D antibody has been shown to ameliorate the neuropathological deficits and behavioral symptoms (111). However, it is still unclear whether SEMA4D inhibition will be beneficial in human HD.

### 4.5 Gangliosides and others

Besides, therapeutic administration of the brain gangliosides, GM1, has also been used to provide neuroprotection in models of neuronal injury and neurodegeneration of Alzheimer’s Disease (AD) (113), Parkinson’s Disease (PD) (114, 115) and HD (116). Furthermore, the intraventricular administration of GM1 showed profound disease-modifying effects across HD mouse models exhibiting varying genetic backgrounds, and there is a reduction in mutant HTT levels after GM1 administration (116). The treatment of R6/2 mice with GM1 slows down the white matter atrophy and body weight loss, while the motor functions have been significantly improved. The administration of GM1 also ameliorated psychiatric-like and cognitive dysfunctions and gait abnormalities. It was also shown that GM1 administration improves psychiatric-like and cognitive dysfunctions in YAC128 mice (116). Prados et al. reported an efficacy of the compound betulinic acid hydroxamate (BAH), a hypoximimetic derivative of betulinic acid, against the striatal HD neurodegeneration. In striatal STHdhQ111/Q111 cells, BAH stabilized HIF-1α protein and protected against mitochondrial toxin-induced cytotoxicity. Pharmacokinetic analyses showed that BAH had a good brain penetrability in 3-nitropropionic acid-treated mouse model with striatal neurodegeneration, improved the clinical symptoms, prevented neuronal loss, decreased reactive astrogliosis and microgliosis, and inhibited the upregulation of proinfammatory markers in the brain (117). Purushothaman et al. also reported the neuropharmacological protective effect of Baicalein (BC) against the Quinolinic Acid (QA)-induced HD-like rat models that displayed the psychological and behavioural changes. This study proved that BC is efficient to revive the level of enzymatic and non-enzymatic antioxidants and mitochondrial complexes by decreasing a number of inflammatory mediators such as Malondialdehyde (MDA), protein carbonyls and Nitric Oxide. It also restores the amount of BDNF and GDNF, thereby preventing the neurophysiological changes (118). Taking together, these findings show that targeting cytokines might assist in resolving neuroinflammation, but evidence suggests that merely suppressing the neuroinflammatory processes would be insufficient to restore functional capacity in HD.

## 5 Concluding remarks

The contribution of neuroinflammation to neurodegeneration has previously been defined. However, the role of CNS and peripheral inflammatory changes in HD remains poorly understood. This is because neuroinflammatory and neuroimmune reactions can be beneficial or detrimental, and there are various interactions between diverse brain cell types and the signaling cascades triggered in HD. Various families of cytokines and cytokine receptors, growth factors, and chemokines influence the apoptotic or survival pathways of neurons and the degree of inflammatory processes in the CNS. It is unclear whether inflammatory changes are caused by neurodegeneration or represent an independent pathological mechanism. Thus, it is important to refine our understanding of these more specific immune and inflammatory mechanisms involved in HD.

Although HD mouse models have been widely used to investigate HD pathogenesis and neuropathology, there are various differences in neurodegenerative pathophysiology between rodent models and clinical patients. In particular, neuronal inflammatory responses were found in HD patients at the early disease stage but were not consistently seen in many mouse models of HD at young ages. Given the lack of obvious neurodegeneration phenotypes in most genetic mouse models, the demand for establishing large animal models to study neurodegenerative diseases is well-appreciated. Investigation of large animal models would be highly valuable for understanding the novel pathogenic mechanisms and identifying neuroinflammation alterations, which may not be uncovered in small animals, though there are challenges and limitations that are largely stemmed from the costly and time-consuming investigation.

In addition, it remains to be investigated whether peripheral immune response and inflammatory alterations mirror the changes and the putative pathways in the CNS in HD. Yet, whether the inflammatory response is an active or a reactive (or both) mechanism in HD pathophysiology remains controversial. Further mechanistic studies are need and would require use of multiple animal models including those large animal models that can more closely mimic the pathological changes in HD patients. Advancing our understanding of the involvement of the immune system in HD pathophysiology would help identify a valid target for new therapeutic interventions to halt the progression of HD.

## Author contributions

QJ and PY wrote the manuscript. X-JL and SL edited the manuscript. All authors contributed to the article and approved the submitted version.

## Glossary

**Table:**

HTT	Huntingtin
HD	Huntington's disease
polyQ	polyglutamine
CNS	Central nervous system
BBB	Blood-brain barrier
YAC	Yeast artificial chromosome
BAC	Bacterial artificial chromosome
LPS	Lipopolysaccharides
IL	Interleukin
TNF	Tumor necrosis factor
MCP-1	Monocyte chemoattractant protein-1
PSD-95	Postsynaptic density protein 95
IBA1	Ionized calcium-binding adapter molecule 1
CX3CL1	C-X3-C motif chemokine ligand 1
QUIN	Quinolinic acid
3-HK	3-hydroxykynurenine
WT	Wild-type
IFN-γ	Interferon gamma
BDNF	Brain-derived neurotrophic factor
NTRK2	Neurotrophic receptor tyrosine kinase 2
GFAP	Glial fibrillary acidic protein
CRISPR	Clustered regularly interspaced short palindromic repeats
KI	Knock-in
CSF	Cerebrospinal fluid
CRP	C-reactive protein
MMP-9	Matrix metalloproteinase-9
CCL	Chemokines (C-C motif) ligand
C1QC	Complement C1q subcomponent subunit C
NK	Natural killer
SEMA4D	Semaphorin-4D
UHDRS	Unified HD rating Scale
LAQ	Laquinimod
EAE	Experimental autoimmune encephalomyelitis
ICV	Intracerebroventricular
PLXNB1	Plexin-B1
PLXNB2	Plexin-B2
AD	Alzheimer's Disease
PD	Parkinson's Disease
BAH	Betulinic acid hydroxamate
HIF-1α	Hypoxia-inducible factor 1-alpha
BC	Baicalein
QA	Quinolinic acid
GDNF	Glial cell derived neurotrophic factor
MDA	Malondialdehyde
